# Coulomb Blockade Effect in Well-Arranged 2D Arrays of Palladium Nano-Islands for Hydrogen Detection at Room Temperature: A Modeling Study

**DOI:** 10.3390/nano10050835

**Published:** 2020-04-27

**Authors:** Mahdi Khaje, Hassan Sedghi, Hadi Goudarzi, Mohammad Taghi Ahmadi, Seyed Saeid Rahimian Koloor, Michal Petrů

**Affiliations:** 1 Department of Physics, Faculty of Science, Urmia University, Urmia 57147, Iran; m.khaje@urmia.ac.ir (M.K.); h.sedghi@urmia.ac.ir (H.S.); h.goudarzi@urmia.ac.ir (H.G.); 2 Institute for Nanomaterials, Advanced Technologies and Innovation, Technical University of Liberec, Studentska 2, 461 17 Liberec, Czech Republic; michal.petru@tul.cz

**Keywords:** Coulomb blockade threshold, hydrogen gas sensor, single-electron tunneling, palladium nanoparticles, room temperature

## Abstract

The fast growth of hydrogen usage as a clean fuel in civil applications such as transportation, space technology, etc. highlights the importance of the reliable detection of its leakage and accumulation under explosion limit by sensors with a low power consumption at times when there is no accumulation of hydrogen in the environment. In this research, a new and efficient mechanism is presented for hydrogen detection—using the Coulomb blockade effect in a well-arranged 2D array of palladium nano-islands—which can operate at room temperature. We demonstrated that under certain conditions of size distribution and the regularity of palladium nano-islands, with selected sizes of 1.7, 3 and 6.1 nm, the blockade threshold will appear in current-voltage (IV) characteristics. In reality, it will be achieved by the inherent uncertainty in the size of the islands in nano-scale fabrication or by controlling the size of nanoparticles from 1.7 to 6.1 nm, considering a regular arrangement of nanoparticles that satisfies single-electron tunneling requirements. Based on the simulation results, the threshold voltage is shifted towards lower ones due to the expansion of Pd nanoparticles exposed to the environment with hydrogen concentrations lower than 2.6%. Also, exploring the features of the presented structure as a gas sensor, provides robustness against the Gaussian variation in nano-islands sizes and temperature variations. Remarkably, the existence of the threshold voltage in the IV curve and adjusting the bias voltage below this threshold leads to a drastic reduction in power consumption. There is also an improvement in the minimum detectable hydrogen concentration as well as the sensor response.

## 1. Introduction

Hydrogen is an essential gas for many industrial processes. In recent years, the consumption of hydrogen has become more popular due to its potential capabilities as a clean fuel and its intrinsic versatility as a reagent. Therefore, a fast and reliable detection of hydrogen leakages or accumulations is required, owing to its explosiveness [1,2]. Many hydrogen gas sensors have been developed and studied, including electrochemical [3,4], conductometric [5,6], Schottky junction [7], field effect [8], optical [9], surface acoustic wave (SAW) [10,11,12], single-electron tunneling-based [13], and bulk acoustic wave [14], which operate based on different mechanisms. The most common operation mechanisms of hydrogen sensors are based on the changes in the electrical resistance, work function, optical properties and electrical current of selective material employed upon the adsorption and desorption of hydrogen gas [15,16,17,18]. The most sensitive hydrogen sensors are able to measure 0.1% hydrogen gas at temperatures between 0–45 °C with a response time of less than 15 s [17]. However, their power consumption is in the order of mW, and therefore developing low power hydrogen gas sensors at concentrations lower than the explosive limit at room temperature (RT) remains a challenging matter [19]. Additionally, there have been some reports of sensors based on complex nanostructures containing palladium and platinum that provide the fastest response speed (2 s for 1% hydrogen concentration) [20,21]. 

Among other mechanisms to detect *H*_2_, the prevalent palladium thin film technique, based on the random arrangement of nano-sized Pd clusters, has been found to be a simple and effective method [17,18]. Upon hydrogen exposure, the resistance of the sensor decreases due to expansion, connecting some of the nanoparticles and finally forming new pathways to pass the electrical current [17,18,22]. However, in the ultra-small grained Pd nano-pattern structure, a surface electron scattering mechanism has been observed, which increases the pathway of electrons in the channel. This mechanism, in contrast to the above mentioned well-known mechanism, increases the electrical resistance of the sensor under the influence of hydrogen gas and acts simultaneously with it [22]. As the size of the Pd nanoparticles decreases and the tunneling phenomenon between adjacent particles occurs, another phenomenon likely to occur is called the single-electron tunneling effect. In fact, the single-electron effect is associated with nano-sized particles (islands) whose small capacitance makes the observation of the phenomenon known as Coulomb blockade possible [23]. The Coulomb blockade is the increase in electrical resistance of a small electronic device involving at least one low-capacitance tunnel junction [24,25]. The IV characteristics of such tunnel junctions exhibit a threshold voltage which the flow of electric current is blocked before (and permitted after). For the conventional single island device, the voltage of this threshold (*V_th_*) depends on the capacitance of the island, which is given as

(1) $$V_{th}=\frac{e}{C},$$

where *e* is the elementary charge of the electron and *C* is the capacitance of the island. Furthermore, the single-electron tunneling and Coulomb blockade only matter if the Coulomb energy (the amount of energy needed to add an electron to the island) is greater than the thermal energy of the electron. Otherwise, the thermal fluctuations will wash out the quantization effects. Therefore, it is necessary that

(2) $$E_{c}=\frac{e^{2}}{2C}>K_{B}T,$$

where *K_B_* is Boltzmann’s constant and *T* is the absolute temperature. To observe the Coulomb blockade phenomenon at room temperature, the capacitance must be in the attofarad range. In this situation, to observe the abrupt Coulomb blockade threshold—the abrupt change in the slope of the IV characteristics at the threshold point in order to the electric current exhibit a significant increase just after this point, while before that it had a negligible amount—the radius of the island must be less than 5 nm and the size of the island must be less than 10 nm. Under these conditions, the phenomenon is visible at room temperature. Given the feasibility of this phenomenon by choosing the appropriate geometry for the islands, even sensors can be suggested which work on this basis. Some of the sensors suggested so far based on the effect of the Coulomb blockade in literature are photodetectors based on the graphene quantum dot array [26], the cryogenic thermometer (up to 60 k) [27], and gas sensors based on porous silicon [28]. 

Palladium nanoparticles form hydride phases which represent size-dependent behavior, where nano-sized islands can absorb different concentrations of hydrogen depending on their size [29]. The lattice parameter of palladium nanoparticles shows a hysteresis loop during the adsorption and desorption of hydrogen, which makes palladium a material of interest for hydrogen gas sensing [30,31]. In practice, to implement the thin film sensor, different sizes and concentrations of palladium nanoparticles can be distributed with high accuracy between two electrodes, which are called drain and source. If the widths of the gaps between the adjacent palladium nanoparticles are small (less than 10 nm), an applied potential difference greater than the Coulomb blockade threshold voltage between the electrodes can transport electrons into or out of the islands by quantum mechanical tunneling [25]. The gaps then form a tunnel barrier with an associated energy barrier. In such a system, single-electron tunneling occurs, which holds the promise of achieving the lowest power consumption in modern electronic devices [32,33]. Hence, the palladium nanoparticles disconnect from each other upon the adsorption of hydrogen gas and the size and widths of the gaps between the nanoparticles change. Theoretically, these distributed palladium nanoparticles can be considered as multiple islands and tunneling barriers. Multiple-island single-electron chains can be modeled using the two parameters of tunneling resistance and capacitance [34]. The multiple-island single-electron chains of tunneling junctions encourage the expansion of a variety of devices due to their smaller size, lower weight, ultra-low power consumption, and high sensitivity. The models of multiple-island single-electron chains are studied by solving master equations or using Monte Carlo methods [34,35,36]. The advantages in contrast with single-island single-electron transistors (SETs)—in similar conditions in terms of dimensions of the islands and tunneling junctions—are ability to stand a higher threshold voltage of Coulomb blockade; lower sensitivity to unwanted effects such as defects, background charges, and uncertainty in the size of the palladium nanoparticles; higher operation temperature; and ease of fabrication [32,37,38]. In this research, we investigated the possibility of single-electron tunneling in well-arranged arrays of palladium nano-islands at room temperature. In this respect, the study of IV characteristics for the emergence and disappearance of the Coulomb blockade by controlling the size of nanoparticles was performed. In addition, the effect of the size distribution of nanoparticles on the sensing behavior of the palladium nanoparticles was inspected by Monte Carlo simulation methods. The response of the sensor to the hydrogen gas flow and consumed power was discussed based on the simulation results. It was expected that the diameter of the individual nano-islands played an important role in the device performance. 

## 2. Assumptions of the Model and Simulation Method

For the Gaussian distribution of the palladium nanoparticles size (diameter):

(3) $$f\left(x\right)=\frac{1}{\sigma_{x}\sqrt{2\pi }}\exp\left(-\frac{{\left(x-\mu_{x}\right)}^{2}}{2\sigma_{x}{}^{2}}\right),$$

where $\mu_{x}$ is the mean diameter of the nanoparticles and $\sigma_{x}$ is their standard deviation. Normally, experimental reports use another parameter instead of $\sigma_{x}$, which is called the full width at half maximum (*FWHM*). This parameter is used to describe the effective width of the Gaussian distribution curve (so-called bell curve). To obtain this parameter, two points of the function in which its value reaches half of the maximum were considered. The distance between those two points is the *FWHM*. For the Gaussian distribution, the relationship between the *FWHM* and the standard deviation was as follows:

(4) $$FWHM=2\sqrt{2ln2}\;\sigma_{x}=2.355\;\sigma_{x}.$$

Evidently, the palladium nanoparticles show a Gaussian distribution, with narrow full widths at half maximum (FWHMs) [29,39]. The extracted FWHMs for the selected sizes of 1.7, 3 and 6.1 nm are shown in Table 1. The physical dimensions of the unit cells in the crystalline lattice of palladium (so-called lattice constant) varied according to the adsorption and desorption of hydrogen gas. This variation was a function of the partial pressure of the hydrogen gas as well as the mean diameter of the palladium nanoparticles. Table 1 lists the lattice constant of palladium nanoparticles of different sizes before and after exposure to hydrogen (partial pressure in torr) [29].

**Figure 1:**
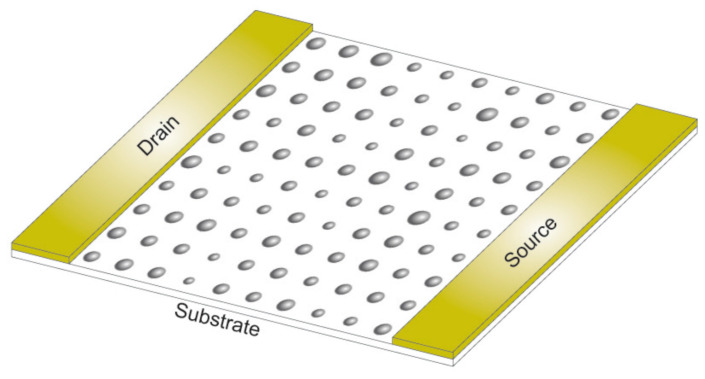
Schematic diagram of 2D arrays of palladium nanoparticles between source and drain electrodes.

We assumed well-arranged 10×10, 15×15 and 20×20 arrays of palladium nanoparticles with a Gaussian distribution for the selected diameters. A schematic diagram of the considered configurations is shown in Figure 1.

**Figure 2:**
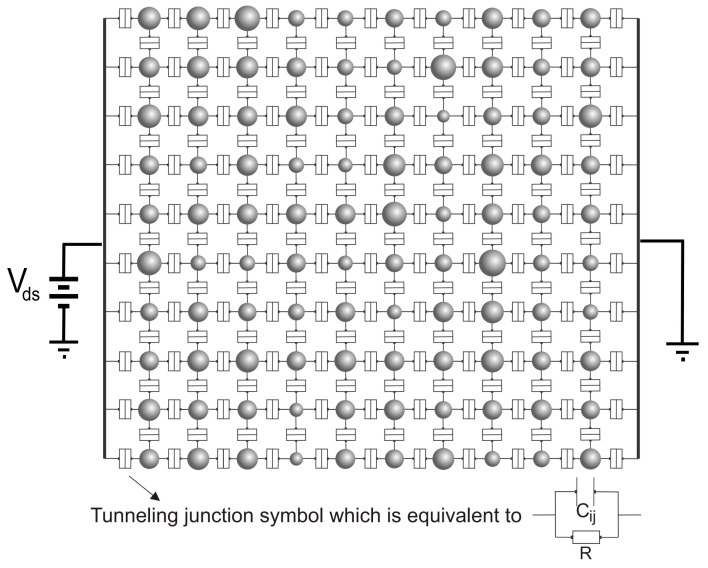
Equivalent circuit of 2D arrays of palladium nano-islands.

The arrays were biased by a source–drain voltage ($V_{ds}$) that was set from 0–20 *V*. Figure 2 shows the equivalent circuits for the assumed configuration of palladium nanoparticles in Figure 1. Using a SIMON2.0 simulator, we investigated the $I_{ds}-V_{ds}$ characteristics of equivalent circuits consisting of palladium nano-islands and tunneling junctions [32,33,40]. The results from the simulations of 10×10, 15×15 and 20×20 arrays are provided in the results and discussion section. The SIMON 2.0 is a single-electron device and circuit simulator which employs the Orthodox theory and Monte-Carlo methods in order to simulate the propagation of electrons in a wide variety of single-electron circuits [41]. Generally, the Orthodox theory assumes a continuous energy spectrum in the conductive islands with negligible cotunneling and tunneling time [25].

The essential parameters in multi-island single-electron arrays are the electrical resistance and capacitance of the tunneling junctions. We assume that the electrical resistance of the tunneling junctions can be described by [42,43]:

(5) $$R\propto e^{\beta L}e^{E_{c}/K_{B}T},$$

where L is the size of the tunneling gap. Here, the activation energy $E_{C}$ is the Coulomb charging energy and β is a system dependent on the tunneling constant given by $\beta =\sqrt{8mU_{0}/h^{-2}}$ [42], with m as the effective mass of an electron. For avoiding simultaneous tunneling, the minimum tunnel resistance of all the tunnel barriers must be much higher than the quantum unit of resistance RQ(R≫RQ=he2∼26.5KΩ), where e is the elementary charge of the electron and h is the Plank constant [29,30].

Here, an analytical method using image charges is implemented to compute junction capacitances $C_{ij}$ between neighboring islands [44,45]:

(6) $$C_{ij}=\frac{4\pi \epsilon_{0}ab}{c}\sinh\left(U\right)\sum_{n=1}^{\infty }\sinh{\left(nU\right)}^{-1},$$

where a and b are the radii of the palladium nano-islands and the dimensionless parameter U is related to *a*, *b* and *c* by $\cosh\left(U\right)=\frac{c^{2}-a^{2}-b^{2}}{2ab}$. Here, *c* is the center–center distance between adjacent palladium nanoparticles.

On the other hand, for the 1D array of islands, the Coulomb blockade threshold voltage is related to the number of junctions (*N*):

(7) $$V_{th}\propto \frac{N}{C_{\Sigma }},$$

where $C_{\Sigma }$ is total capacitance of the chain. This was achieved under the above conditions for tunnel resistance. The linear relationship (shown above) between the threshold voltage ($V_{th}$) and the number of junctions (N) has been detected in 2-dimensional systems [46,47].

It is well known that palladium nanoparticles form hydride phases which absorb a substantial quantity of hydrogen within their crystal lattice. At room temperature, palladium hydrides may contain two crystalline phases, α and β (sometimes called $\alpha^{\prime }$). Therefore, the lattice parameter of palladium nanoparticles shows a hysteresis loop during the adsorption and desorption of hydrogen. Bridget et al. [29] have investigated the α and β phases in bare palladium upon hydrogen gas and obtained the variation in the lattice parameter versus the hydrogen pressure. The results were used for the lattice parameters of the selected diameters 1.7, 3 and 6.1 nm in this study. The expansion of the individual nanoparticles increased the area occupied by each nanoparticle and this in turn decreased the average size of the tunneling gap. Thus, according to Equations (1) and (2), the resistance and capacitance of tunneling junctions change.

Owing to the expansion of the palladium nano-islands, the following steps were employed in order to calculate the new resistance and capacitance of the tunneling junctions: (1) A change (Δa) occurs in the lattice parameter (*a*) due to the change in the external hydrogen pressure. (2) The variation (Δa) in the lattice parameter causes the change (Δd) in diameter (*d*) of the palladium nanoparticles (Δd=(Δa/a)d). (3) The expansion of individual nanoparticles increases the area occupied by each nanoparticle, which decreases the average size of the tunneling gap (*L*). For an array whose number of nanoparticles is equal to *p* in length, the change in *L* is given by

(8) $$\Delta L=-\frac{pd}{L}\Delta \alpha .$$

In reality, hydrogen will slightly change the band in palladium nano-islands and accordingly it will change beta in expression (1). One can find that this effect is not important in the IV characteristics, and therefore only the change in the tunneling gap (*L*) was considered. Furthermore, the temperature of 300 K (the temperature for detecting hydrogen) was used in the simulations. After setting some parameters like temperature, the mode of simulation, the resistance and capacitance of tunneling junctions, the IV characteristics were inspected.

## 3. Results and Discussion

**Figure 3:**
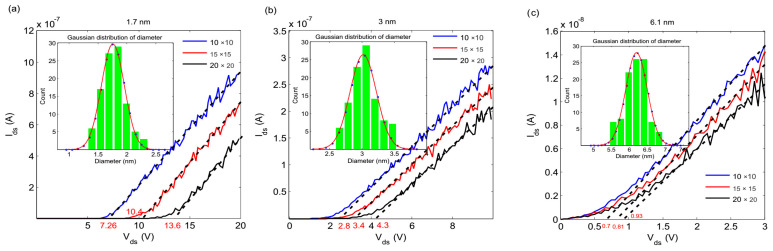
The $I_{ds}-V_{ds}$ characteristics of arrays for (**a**) 1.7, (**b**) 3, and (**c**) 6.1 nm nano-islands, simulated by spreading the array size at room temperature.

The variations in $I_{ds}-V_{ds}$ for well-arranged arrays with 1.7, 3 and 6.1 nm nano-islands at room temperature are shown in Figure 3. The IV characteristics are plotted for 10×10, 15×15 and 20×20 square arrays, along with the size distribution of the palladium nanoparticles. The Coulomb blockade is clearly observed. At bias voltages above the threshold value, the dc curve gradually approaches to the offset linear asymptotes with increasing applied voltage. The extrapolation of the linear regions results in $V_{th}$, which represent the basic conceptual principle of threshold voltage [48]. The obtained $V_{th}$ for the arrays are listed in Table 2. It is remarkable that the current suppression region for the arrays decreases with the increasing mean diameter of the nanoparticles. According to relation (3), this reduction in the current suppression region is probably due to the increase in the total capacitance of the array.

The effect of increasing the length of the arrays on the Coulomb blockade threshold can be seen in Figure 3. According to the results, the increase in the Coulomb blockade threshold voltage with the length of the array is linear. The simulation results show that it is the length of the array that determines the value of the Coulomb blockade threshold voltage. The total resistance of the device for voltages above the Coulomb blockade threshold is directly proportional to the length of the device and inversely proportional to the width.

The possibility of using these arrays as single-electron tunneling-based hydrogen sensors was investigated. The IV characteristics of the arrays were inspected upon their exposure to hydrogen gas. In this study, the hydrogen pressure of 20 torr was selected for investigation. The lattice parameters of 1.7, 3 and 6.1 nm palladium nanoparticles were extracted for 20 torr pressure and are listed in Table 1.

**Figure 4:**
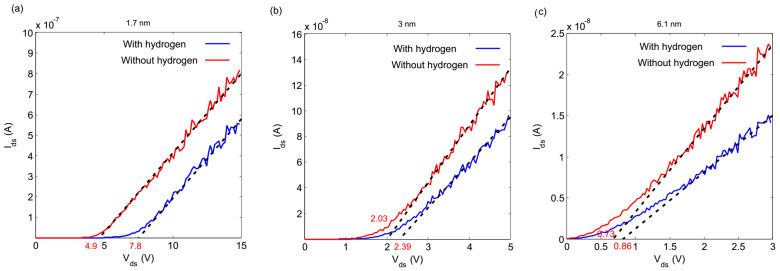
$I_{ds}-V_{ds}$ characteristics of 10 *×* 10 arrays for (**a**) 1.7, (**b**) 3, and (**c**) 6.1 nm palladium nano-islands before and after exposing to hydrogen, simulated at *T* =300 *K*. (Dash line: fitted exponential curve).

The changes in tunneling resistance and capacitance were calculated for each tunneling junction according to the procedure mentioned in the assumptions of the model. Then, the IV characteristics with new parameters were plotted. Figure 4 shows the IV characteristics of the arrays before and after exposure to hydrogen. The IV characteristics separated before and after exposure to hydrogen gas, as can be seen in Figure 4. This happened because the expansion of individual nanoparticles increases the area occupied by each nanoparticle and decreases the total resistance of the arrays, as well as increasing their capacitance. 

**Figure 5:**
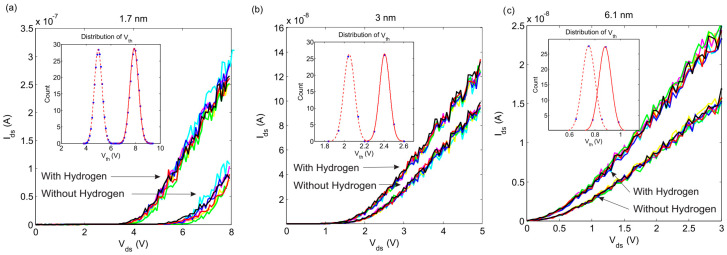
Effect of the Gaussian distribution on the threshold voltage for 10 × 10 arrays of (**a**) 1, (**b**) 3, and (**c**) 6.1 nm Pd nano-islands at room temperature. (Inset images show the distribution of the calculated threshold voltage before (solid) and after (dash) H_2_ exposure).

The obtained *V_th_* for the IV characteristics after the exposure to hydrogen are also shown in Table 2. The decrease in the Coulomb blockade threshold was related to the increase in the total capacitance of the arrays. The simulated nanoparticles had inherent randomization in their size, therefore we randomized the diameter of the individual nano-islands according to the Gaussian distribution shown in Figure 3. Accordingly, we carried out 100 simulations in which the diameter of any individual nano-island in the arrays was changed randomly in each simulation. The IV characteristics were plotted each time and the Coulomb blockade threshold was observed for each array. The results of these simulations for 10×10 arrays are shown in Figure 5, which indicates that the IV characteristics overlap and the Coulomb blockade threshold does not change. These results show that the IV characteristics of these arrays were not dependent on the diameter of the individual nano-island. In fact, whilst the size of the nano-islands obeyed Gaussian distribution, the size of the individual nano-island was not important. In this study, the Gaussian distribution obtained for the Coulomb blockade threshold voltages before and after being exposed to the hydrogen gas is shown in the inset of Figure 5. The obtained distributions do not overlap and there is enough of a split between them, which assures that the well-arranged arrays of palladium nanoparticles can be used as a hydrogen sensor.

**Figure 6:**
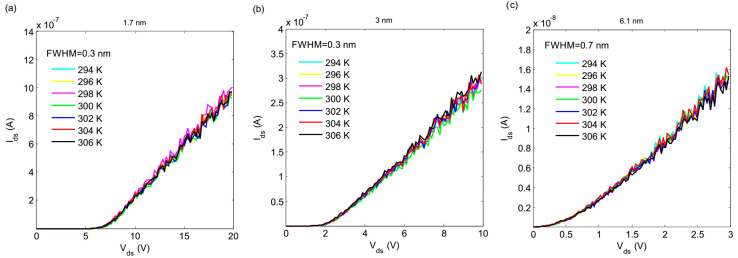
Effect of temperature variation on the Coulomb blockade threshold voltage of arrays for (**a**) 1.7, (**b**) 3, and (**c**) 6.1 nm palladium nano-islands.

In addition, another similar simulation was carried out at different temperatures, in which the results imply the robustness of the sensor against variations in temperature. The effect of temperature on the IV characteristics is shown in Figure 6. The sensitivity of the single-electron tunneling effect to temperature variation depends on the size of the islands that are used in the arrays. For sub-10-nm palladium nanoparticles at room temperature, the Coulomb blockade threshold is abrupt and there is no sub-threshold current (especially for smaller nanoparticles, as can be seen in Figure 6a,b). When the temperature increases, the sharpness of the Coulomb blockade threshold decreases slowly. As a result, the IV characteristics exhibit a sub-threshold current. This effect increases the power consumption of the device in idle mode (when there is no leakage in the environment) and decreases the sensor response. It is shown that the phenomenon of sub-threshold leakage is less severe in smaller islands, and arrays with a smaller island size can exhibit very low sub-threshold leakage even at higher temperatures. 

An important factor in hydrogen sensors is their response, such that in thin palladium film, adjacent nanoparticles have to connect to each other in order to detect the hydrogen gas. However, in tunneling mechanism they do not need to connect to adjacent nanoparticles in order to pass the electrical current and detect the hydrogen gas. Typically, metal hydrogen sensors operate by resistance variation sensation. By hydrogen absorption, the palladium hydride showed a higher electrical resistance in comparison to the palladium [3]. The variation in the total resistance of the arrays at voltages greater than $V_{th}$ can be used to detect hydrogen. Indeed, according to the results obtained, the Coulomb blockade threshold does not change upon variations in temperature and size randomization of nano-islands. Therefore, considering the shift of $V_{th}$ upon hydrogen adsorption, the Coulomb blockade threshold variations can be used as the sensing parameter of hydrogen gas. With the ideal gas approximation and using Dalton’s law, the volume concentration of hydrogen gas ($\sigma_{H_{2}}$) in standard conditions is calculated from

(9) $$\sigma_{H_{2}}\left(\%\right)=\frac{P_{H_{2}}\left(torr\right)}{P_{ATM}\left(torr\right)}=\frac{P_{H_{2}}\left(torr\right)}{760}\;,$$

where $P_{H_{2}}$ is the partial pressure of hydrogen gas (torr), and $P_{ATM}$ is the atmosphere pressure. At the selected partial pressure of 20 torr of hydrogen gas, the proposed sensor showed the capability of detecting 2.6% hydrogen gas in air, which is less than flammability and explosibility concentration of hydrogen gas (4%) [17,49]. Therefore, it is highlighted that the detection of lower concentrations of hydrogen is possible through a proper selection of the suitable size of palladium nano-islands and distance between them. On the other hand, since this sensor is based on single-electron tunneling between palladium nano-islands, the sensor is actually an ultra-low power consumption sensor. 

**Figure 7:**
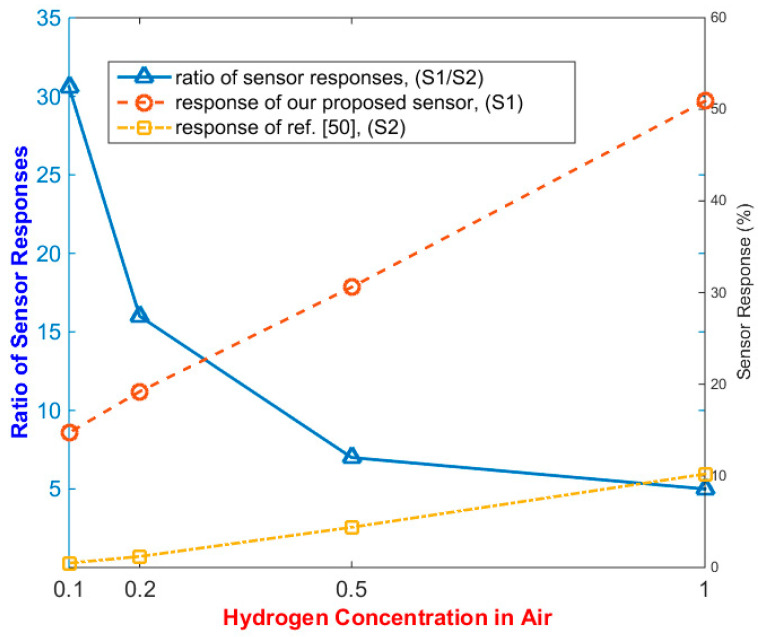
The response of our proposed hydrogen sensor based on 1.7 nm nanoparticles in comparison with the reference [50] in volume concentrations 0.1%, 0.2%, 0.5%, and 1% of hydrogen in ambient air, and the ratio of the response of our sensor to that of the reference [50].

The response of our sensor (S_1_), which was proposed based on an array of 1.7-nm-size nanoparticles, was examined when the sensor was biased exactly at the Coulomb blockade threshold point. In this situation, the hydrogen concentration varied from 0.1% to 1%. Figure 7 displays the response of our proposed sensor (S_1_) in comparison with another palladium-based sensor (S_2_) which uses palladium nanoparticles [50]. Since the electrical resistance of our sensor at the threshold point changed dramatically, the shift of the threshold to lower voltages severely reduced the electrical resistance. Despite decreasing the response of the sensor by lowering the concentration of hydrogen gas, our sensor response (S_1_) exhibited a lower reduction rate in comparison with (S_2_). In other words, by reducing the concentration of hydrogen gas—despite the declining trend in the response of both sensors—the ratio of the response of our sensor (S_1_) to (S_2_) is increasing.This suggests that our sensor could potentially be used for hydrogen gas leak detection, which needs a detection of concentration range, typically from 0.1% to less than 5% [18]. Since, in the subthreshold region, the IV characteristics of our sensor exhibited exponential behavior, it promised higher amounts of response due to the higher variation in electrical resistance in this region. In this situation, a higher amount of electrical resistance can cause a lower power consumption when there is no leakage (even less than 1 nW). However, the increasing static electrical resistance may raise the white noise in the sensor, which will reduce the minimum detectable concentration.

## 4. Conclusions

Hydrogen gas as a clean energy is becoming part of transport applications of civil industry, resulting in the fast growth of its novel production and consumption. Since hydrogen is very explosive, the fast and reliable detection of its leakage and accumulation under the explosion limit is an important design criteria for gas-consuming systems. Additionally, the power consumption of hydrogen sensors in idle mode (when there is no hydrogen leakage in environment) is an essential figure of merit. In this study, the possibility of hydrogen gas sensing was shown using the Coulomb blockade effect in well-arranged arrays of palladium nano-islands at room temperature. The single-electron tunneling threshold in the IV characteristics of arrays with selected sizes of palladium nanoparticles arranged in a regular lattice was observed. The results indicate that an island size of 6.1 nm is an upper limit for keeping the Coulomb blockade existence in arrays of palladium nano-islands at room temperature. The Monte Carlo simulation method—which we used on the arrays of palladium nanoparticles with sizes obeying Gaussian distribution—showed that these arrays are resistant to the size variation in nanoparticles, as presented in this research. Furthermore, it depicted a measuring concentration of 2.6% with an expected response time of less than 15 s and power consumption of less than 1nW power when there is no leakage in the environment. The comparison of our proposed sensor with another one (which was also designed based on palladium nanoparticles), suggests that our sensor could potentially be used for hydrogen gas leak detection, which needs a detection in the concentration range of 0.1% to less than 5%.

## Figures and Tables

**Table 1:** Extracted parameters from the literature [29,39] that were used in the simulations.

Mean diameter of nanoparticles, nm	1.7	3	6.1
Full width at half maximum, nm	1.1	0.3	0.7
Lattice constant before exposing to hydrogen, °A	3.9167	3.9167	3.90
Lattice constant after exposing to hydrogen (20 torr), °A	3.9232	3.9217	3.92
Tunneling gap between adjacent nano-islands, nm	2	2.25	3

**Table 2:** *V_th_* calculated from the extrapolation of linear regions to zero.

Mean Diameter of Nanoparticles, nm	1.7	3	6.1
*V_th_* for 10 × 10 arrays of nano-islands before exposing to hydrogen, V	7.26	2.8	0.83
*V_th_* for 10 × 10 arrays of nano-islands after exposing to hydrogen (20 torr), V	4.9	2.03	0.73
*V_th_* for 15 × 15 arrays of nano-islands, V	10.4	3.4	0.81
*V_th_* for 20 × 20 arrays of nano-islands, V	13.6	4.3	0.93
